# ErbB4 precludes the occurrence of PTSD-like fear responses by supporting the bimodal activity of the central amygdala

**DOI:** 10.1038/s12276-024-01365-1

**Published:** 2024-12-02

**Authors:** Kibong Sung, Min-Jae Jeong, Taesik Yoo, Jung Hoon Jung, Sumin Kang, Jong-Yeon Yoo, Hyun Jin Kim, Kyunghyun Park, Jung Hyun Pyo, Hyun-Yong Lee, Noah Koo, Soo-Hee Choi, Joung-Hun Kim

**Affiliations:** 1 Department of Life Sciences, Pohang University of Science and Technology (POSTECH), Nam-gu, Pohang, Gyeongbuk 37673 Republic of Korea; 2 College of Pharmacy, Keimyung University, 1095 Dalgubeoldaero, Dalseo-gu, Daegu 42601 Republic of Korea; 3 Department of Psychiatry, Seoul National University College of Medicine, Seoul, 03080 Republic of Korea; 4 Institute of Convergence Science, Yonsei University, Seoul, 03722 Republic of Korea

**Keywords:** Amygdala, Fear conditioning, Stress and resilience, Genetics of the nervous system

## Abstract

Post-traumatic stress disorder (PTSD) often arises after exposure to traumatic events and is characterized by dysregulated fear responses. Although the associations of erb-b2 receptor tyrosine kinase 4 (ErbB4) with various neuropsychiatric diseases, including schizophrenia and bipolar disorder, have been widely examined, the physiological roles of ErbB4 in PTSD and fear responses remain unclear. Using Cre-dependent ErbB4 knockout (KO) mice, we observed that PTSD-like fear behaviors emerged in ErbB4-deficient mice, particularly in inhibitory neurons. Specifically, the loss of ErbB4 in somatostatin-expressing (SST^+^) neurons was sufficient to induce PTSD-like fear responses. We also adopted the CRISPR/Cas9 system for region-specific KO of ErbB4, which revealed that ErbB4 deletion in SST^+^ neurons of the lateral division of the amygdala (CeL) caused elevated anxiety and PTSD-like fear generalization. Consistent with its physiological role, ErbB4 expression was diminished in CeL^SST^ neurons from mice that exhibited PTSD-like phenotypes. While fear On and Off cells identified in the CeL displayed distinct responses to conditioned and novel cues, as previously shown, the selectivity of those On and Off cells was compromised in SST^ErbB4-/-^ and stressed mice, which displayed strong fear generalization. Therefore, the bimodal activity that CeL On/Off cells display is likely required for proper discrimination of fearful stimuli from ambient stimuli, which should be sustained by the presence of ErbB4. Taken together, our data substantiate the correlation between PTSD-like fear responses and ErbB4 expression in CeL^SST^ neurons and further underscore the functional effects of ErbB4 in CeL^SST^ neurons, supporting the bimodal responses of CeL neurons.

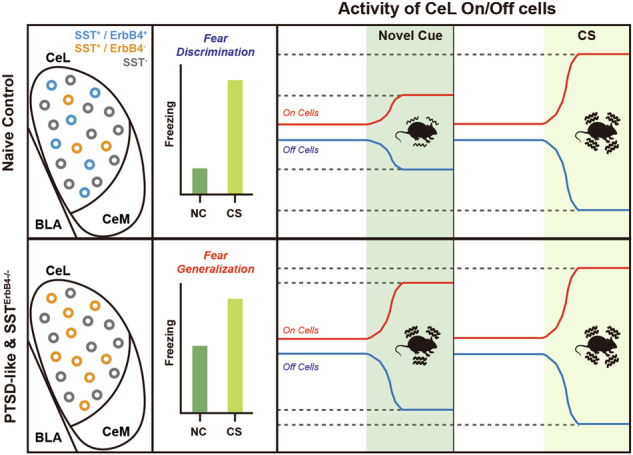

## ErbB4 deficiency triggers PTSD-like fear responses in mice

Post-traumatic stress disorder is a mental health condition that can develop after experiencing traumatic events. Researchers tried to understand the biological basis of PTSD using animal models. The researchers investigated the role of a protein called ErbB4 in fear responses related to PTSD. They used mice to study how deleting ErbB4 in specific brain cells affects fear behavior. They focused on somatostatin(SST)-expressing neurons in a brain region called the central amygdala, which is involved in processing fear. The study involved genetic modification, behavioral tests, and in vivo recording to observe changes in fear responses. The findings showed that removing ErbB4 from SST+ neurons led to increased anxiety and generalized fear, like PTSD symptoms, with specific alteration of neuronal activity. This suggests that ErbB4 helps regulate fear responses, and its absence may contribute to PTSD-like behaviors.

This summary was initially drafted using artificial intelligence, then revised and fact-checked by the author.

## Introduction

Post-traumatic stress disorder (PTSD) is a maladaptive psychiatric disorder that can often occur following exposure to traumatic events^1–3^. PTSD has a lifetime prevalence rate of 3.9% in the total population and 5.6% among individuals exposed to trauma^4^. Various rodent models have been employed to explore the etiological basis of PTSD in laboratory settings^5–7^. Such animal models should fulfill the criteria for PTSD outlined by the Diagnostic and Statistical Manual of Mental Disorders, Fifth Edition, encompassing physiological and behavioral alterations^8,9^. The endophenotypes of PTSD that animal models exhibit include generalized fear responses to ambient cues and resistance to exposure therapy (extinction)^8,10,11^.

The central nucleus of the amygdala (CeA) plays critical roles in physiological and behavioral responses to fearful stimuli, such as the integration of external and internal sensory information and the orchestration of innate and adaptive behaviors^12–14^. In particular, the physiological and functional roles of the lateral division of the CeA (CeL) have been shown for fear learning^15,16^ and extinction^17,18^. Interestingly, CeL neurons mainly consist of somatostatin-expressing (SST^+^) and protein kinase C-δ-expressing (PKC-δ^+^) neurons^19^; SST^+^ neurons in the CeL are considered to primarily mediate anxiety^20,21^ and defensive behaviors^13^.

Previous in vivo recordings revealed that CeL neurons display bimodal activity in response to conditioned cues (fearful stimuli), indicating the activation of a subset of neurons (On cells) and the inhibition of another subset (Off cells)^16,22^. Interestingly, a majority of On cells are SST^+^ neurons, whereas PKC-δ^+^ neurons are mainly Off cells^23–25^. While the activity of CeL On/Off cells has been monitored during fear extinction^22^, the responses that On/Off cells display during fear generalization and their physiological roles have not yet been thoroughly examined.

ErbB4, a receptor tyrosine kinase in the epidermal growth factor receptor family, becomes activated upon binding with its ligand, neuregulin-1 (NRG1)^26^. In the central nervous system, ErbB4 is expressed in inhibitory interneurons^27–29^. ErbB4-NRG1 signaling plays a determining role in the appropriate development of interneurons and their synapses^30–32^. For example, the inhibition or activation of ErbB4-NRG1 signaling affects the generation and maturation of both inhibitory and excitatory synapses^30,31,33^. Considering the physiological actions that ErbB4 performs in the development and maintenance of inhibitory synapses, its well-established correlation with neuropsychiatric disorders, such as schizophrenia and bipolar disorder, is noteworthy^26,34,35^. Previous reports have indicated that ErbB4 expressed in parvalbumin (PV)-expressing neurons affects fear expression and extinction^36^. However, whether and how ErbB4 in various GABAergic neurons can modulate fear memory, particularly PTSD-like traits such as fear generalization and deficits in fear extinction, remains elusive.

Here, we examined the physiological and behavioral consequences of cell type-specific ErbB4 deletion on the regulation of learned fear. We also explored any potential correlation between ErbB4 expression in SST^+^ neurons and the occurrence of PTSD-like behaviors by assessing ErbB4 expression in the CeL of mice displaying PTSD-like fear behaviors. After validating the intrinsic bimodal responses of individual CeL neurons specific to fearful stimuli through in vivo single-unit recordings as previously shown^16,22^, we discovered that the activity specificity of On and Off cells in the CeL was compromised in CeL SST^ErbB-/-^ and stressed mice, both of which exhibited pronounced fear generalization. Collectively, our data provide a novel perspective on the roles of ErbB4 and CeL activity in PTSD-like responses, indicating that ErbB4 deletion in SST neurons can trigger PTSD-like fear responses by affecting CeL neuronal activity.

## Materials and methods

### Subject mice

The mice were housed under a light/dark cycle (12/12 h) with food and water *ad libitum*. Male adult C57Bl/6J (Jackson Laboratory, MA, US), B6;129-Erbb4^tm1Fej^/Mmucd (MMRRC, 010439-UCD), Tg(Dlx5a-cre)1Mekk/J (JAX #008199), Sst-IRES-Cre (JAX #028864), B6;129P2-Pvalb^tm1(cre)Arbr^/J (JAX #008069), Vip^tm1(cre)Zjh^/J (JAX #010908), and B6J.129(B6N)-Gt(ROSA)26Sor^tm1(CAG-cas9*,-EGFP)Fezh^/J (JAX #026175) mice were used. All procedures for the animal experiments were approved by the Institutional Animal Care and Use Committee of POSTECH, Korea, and were performed in accordance with the relevant guidelines.

### Behavioral tests and analysis

Fear conditioning and tests were conducted in accordance with our established protocols^6^ and in three different contexts. Context A consisted of a chamber (17.75 cm × 17.75 cm × 30.5 cm) constructed of aluminum and plexiglass walls with infrared lighting (Coulbourn, MA, USA) and a metal grid floor connected to a shock generator (Model H13–15; Coulbourn). Context B was a white acrylic chamber placed inside the Context A chamber, with a mild peppermint scent, aspen bedding, and a light on. Context C was a black acrylic chamber placed inside the Context A chamber, with a 1% acetate scent, corncob bedding, and a light on.

Habituation and fear conditioning were conducted in Context A. Before fear conditioning, the mice underwent 5 min of habituation for 2 consecutive days. During the fear conditioning session, the mice were allowed 2 min for acclimation, followed by 4 presentations of a tone (conditioned stimulus, CS: 10 kHz, 80 db, 30 s) paired with co-terminating electric foot shocks (unconditioned stimulus, US: 0.4 mA, 0.5 s) at pseudorandom intertrial intervals of 60–120 s. The generalization test was performed in Context B; after acclimation (3 min), the mice received three presentations of the novel cue (NC: 2 kHz, 80 db, 30 s) followed by three presentations of the CS. Extinction training was conducted in Context C; after acclimation (2 min), the mice received 30 presentations of the CS without the US at 5 s intertrial intervals. The extinction memory test was also conducted in Context C, which involved three presentations of the CS with 90 s intertrial intervals following acclimation (2 min).

Freezing responses were evaluated using FreezeFrame software (Coulbourn) by analyzing video recordings across all sessions. Freezing responses were measured as the duration of periods without movement longer than 1 s and are presented as a percentage. The generalization index was defined as the ratio between the freezing duration in response to a novel cue and freezing duration in response to the CS in fear generalization sessions and was calculated as [NC freezing/CS freezing]^37^. The extinction memory deficit index was also defined as the ratio of the freezing duration in response to the CS in the extinction memory session and freezing duration in response to the CS in the fear generalization session and was calculated as [CS freezing _(extinction memory test)_/CS freezing _(generalization test)_].

The elevated plus maze (EPM) apparatus consisted of four arms (30 cm long and 5 cm wide) constructed from white Plexiglas and was raised 60 cm above the floor. Two arms were enclosed by 30-cm-high black Plexiglas walls, and the other two open arms had no walls. The mice were allowed to explore the maze for 15 min, and their behavior was recorded using a video camera. EPM test data were analyzed using SMART 3.0 (Panlab, Cornellà, Spain).

### Stress exposure

All procedures for stress exposure were conducted 1 week prior to the behavioral tests. The exposure protocol by which stresses were applied and then PTSD-like and resilient groups were distinguished was used in accordance with our established protocols^6^. The mice were subjected to 1 hour of restraint stress along with 60 inescapable tail shocks (1 mA, 1 s) administered at pseudorandom intervals of 30–90 s using a shock generator (SCITECH, Seoul, Korea).

The single prolonged stress (SPS) procedure was conducted similarly to what was previously used^38^. Briefly, the SPS mice were restrained for 2 h, followed by a 10 min forced swim in water. After completing the forced swim test, the mice were exposed to ether vapor until they lost consciousness (exposure <5 min).

### DNA construction and virus production

We employed a sgRNA design tool (ATUM BIO CRISPR gRNA design tool) to produce ErbB4-targeting sgRNA sequences (sgErbB4v1: AGCCTCCAGCACATTCTCGATGG; sgErbB4v2: TTGGGGACATTGAGTAACGCAGG; sgErbB4v3: TTGGGGCAAATATCGGTGCAAGG; sgControl: GGGTCTTCGAGAAGACCT). These sequence oligomers were inserted into the BbsI enzyme site of the pSp-Cas9(BB)-2A-GFP vector (PX458; Addgene plasmid #48138) for the transcription of spCas9 and the sgRNA with a gRNA scaffold. Either sgErbB4 or control sequences were inserted into the BbsI enzyme site of the pSMART vector (Addgene plasmid #80427) for transcription of the sgRNA with the gRNA scaffold under the U6 promoter. These U6-sgErbB4 (or control) gRNA scaffold constructs were subsequently cloned and inserted into the MluI enzyme site of the pAAV-EF1a-DIO-mCherry vector.

N2A cells were cultured in Dulbecco’s modified Eagle’s medium containing 10% fetal bovine serum and 1% penicillin/streptomycin in an incubator with a humidified environment of 5% CO_2_ and 95% O_2_ at 37 °C. N2A cells were transfected with CMV-Cas9-EGFP-U6-sgRNA-gRNA scaffold vectors using Lipofectamine 3000 (Thermo Fisher, MA, US) and incubated for 2 additional days after transfection. After confirming transfection via fluorescence, N2A cells were harvested, and total RNA was extracted using an RNA extraction kit (easy-spin, iNtRON Biotechnology, Seongnam, Korea). cDNA was synthesized from total RNA using iScript Reverse Transcriptase (Bio-Rad, CA, US). Quantitative RT‒PCR was performed using the PowerSYBR Green protocol (Thermo Fisher). The fold change in ErbB4 expression was calculated with the delta-delta CT method^39^ using beta-actin as a reference gene (primers for ErbB4 [forward 5′-AGGGGTGTAACGGTCCCACT-3′ and reverse 5′-TCCAATGACTCCGGCTGCAA-3′], and primers for beta-actin [forward 5′-GACCTCTATGCCAACACAGT-3′, reverse 5′-AGTACTTGCGCTCAGGAGGA-3′]).

The adeno-associated virus (AAV) was produced in accordance with our established protocols^40,41^. Briefly, pAAV-U6-sgErbB4 (or sgControl) was cotransfected with AAV helper plasmids (delta-F6) and capsid (serotype 5) into HEK293T cells at an equal molar ratio using Lipofector Q (AptaBio, Yongin, Korea). AAV particles were isolated and purified by iodixanol-gradient ultracentrifugation. The resulting AAV particles were washed and concentrated using an Amicon filter (100 K, Millipore, MA, US) to achieve at least 2.0 × 10^12^ gc/ml.

### Stereotaxic surgery

For virus injection, the mice were anesthetized via an intraperitoneal injection of a mixture of ketamine and xylazine (100 mg/kg and 14 mg/kg, respectively). The mice were fixed in a stereotaxic frame using ear bars (Kopf Instruments, CA, USA). The coordinates for the CeL were −1.3 mm anteroposterior, ±2.9 mm mediolateral, and −4.6 mm dorsoventral. The total injection volume was 50 nl for the CeL. The virus solution was infused at 1 nl/s using a Nanoject III (Drummond Scientific, PA, USA).

For the chronic implantation of electrodes, the mice were anesthetized with isoflurane using SomnoSuite (Kent Scientific, CT, USA). Stainless steel screws were implanted into the skull for electrode fixation. Each bundle of electrodes, consisting of 16 individually insulated nichrome wires (with a diameter of 15 μm and impedance of 70–120 KΩ; A-M Systems, WA, USA), was attached to the electrode guides. Two electrodes were connected to a 36-pin dual-row male nano connector (Omnetics, MN, USA). The connectors were referenced and grounded via four insulated silver wires (127 μm in diameter; A-M Systems). The electrodes were slowly placed into the CeL (1–5 μm/s) using a manipulator (Scientifica, Uckfield, UK), and the reference and ground wires were placed into the cerebellum. Electrodes and connectors were firmly fixed on the skull with Superbond (Sun Medical, Moriyama, Japan). The mice were allowed to recover for 21 days after surgery.

### In vivo unit recordings and analysis

For in vivo recordings, the mice were briefly anesthetized with isoflurane (isoflurane exposure <20 s) and connected to a Cereplex μ-headstage (Blackrock Microsystems, UT, USA), which relayed signals to Cereplex Direct (Blackrock Microsystems). The mice were allowed a minimum of 5 min to recover from the brief anesthesia. The acquired signals were bandpass-filtered at 200 Hz and 5 kHz for spike detection. Single-unit spike sorting was performed using a Blackrock offline spike sorter (Blackrock Microsystems). Principal component scores were automatically calculated for unsorted waveforms using the Blackrock offline spike sorter and represented in three-dimensional principal component spaces. Clusters containing comparable valid waveforms were manually identified^42^.

We classified On and Off cells using a previously reported method in which firing rates were z score transformed before assessing responses to stimuli^16^. We adapted this classification method because the stimuli in this study were tones and not pips. The response to stimuli was calculated based on the baseline firing rate during the acclimation period. Units with an average z score of more than 1 or less than −1 during the presentation of the CS and NC were classified as On and Off cells, respectively.

### Ex vivo electrophysiology

Acute brain slices were prepared in coronal sections. All the chemicals used for the electrophysiological experiments were purchased from Sigma, unless specified otherwise. Whole-cell patch-clamp recordings were obtained with a MultiClamp 700B amplifier (Molecular Devices, CA, USA) in artificial cerebrospinal fluid (aCSF) containing 119 mM NaCl, 2.5 mM KCl, 1 mM MgSO_4_, 1.25 mM NaH_2_PO_4_, 26 mM NaHCO_3_, 10 mM d-glucose and 2.5 mM CaCl_2_, equilibrated with 95% O_2_ and 5% CO_2_ (pH 7.3–7.4) at room temperature (RT). Recording electrodes (4–8 MΩ) were filled with an internal solution containing 130 mM CsMeSO_4_, 8 mM NaCl, 10 mM phosphocreatine, 10 mM HEPES, 0.5 mM EGTA, 2 mM MgATP, 0.2 mM NaGTP, and 5 mM QX-314 at pH 7.2, adjusted with CsOH in a voltage‒clamp configuration. Miniature IPSCs were recorded at +10 mV holding potential in the presence of tetrodotoxin (1 µM, Tocris, Bristol, UK), CNQX (10 µM) and DL-AP5 (25 µM), and series resistance (10–30 MΩ) was monitored.

### RNAscope® fluorescence in situ hybridization (FISH) and imaging

An RNAscope® multiplex fluorescent assay (320850, Advanced Cell Diagnostics, CA, USA) was used to visualize mRNA probes for SST (404631), ErbB4 (318721), and mCherry (431201) according to the manufacturer’s instructions. Mouse brains were rapidly frozen in liquid nitrogen immediately after extraction and sectioned into 15-μm slices using a cryostat (Leica, Wetzlar, Germany). These slices were mounted onto glass slides and subjected to a series of steps, including fixation, dehydration, hydrogen peroxide treatment, and protease treatment. Those slices were subsequently incubated with the mRNA probes for 2 h at 40 °C and then mounted with UltraCruz mounting medium (Santa Cruz Biotechnology, TX, USA) for imaging.

A laser scanning confocal microscope (FV3000, Olympus, Hachioji, Japan) was used for the acquisition of fluorescence images. The numbers of DAPI-stained and SST- and ErbB4-expressing cells were manually counted.

### Quantification and statistical analysis

All the statistical analyses were performed with PRISM 10 (GraphPad Software, MA, US). The datasets were tested for normality with the Shapiro‒Wilk test. When datasets followed a Gaussian distribution, ANOVA or Student’s *t*-tests were employed. The Mann‒Whitney *U*-test was employed for comparisons of non-Gaussian datasets. All the results are shown as the means ± standard errors of the means (SEMs), with *p* values as follows: *p < 0.05, **p < 0.01, ***p < 0.001, and ****p < 0.0001.

## Results

### Deletion of ErbB4 in inhibitory neurons affects the normal regulation of fear expression

**Fig. 1:**
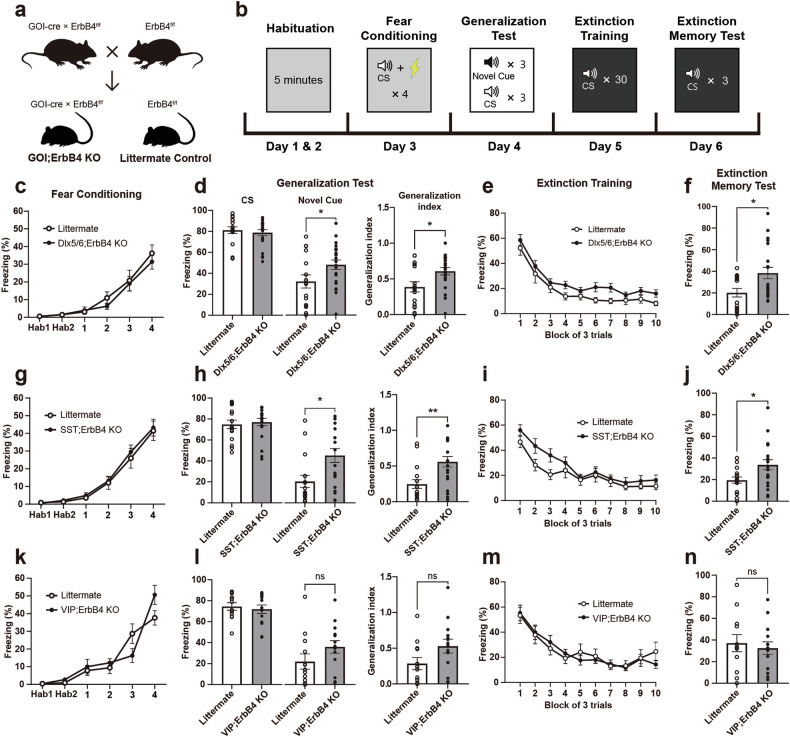
Deletion of ErbB4 in inhibitory neurons, especially SST neurons, affects the expression of learned fear. **a** A schematic depicting the generation of cell type-specific ErbB4 KO mice. **b** Schematic showing the timeline of the experiments. **c**–**f** Auditory fear conditioning and behavioral data for Dlx5/6;ErbB4 KO (*n* = 21) and littermate control (*n* = 16) mice. **c** Basal freezing levels during habituation and freezing levels to the CS during fear conditioning (two-way repeated measures (RM) ANOVA followed by Sidak’s post hoc test, *p* > 0.05). **d** Mean freezing levels in response to the CS (left panel, Mann–Whitney *U*-test, *p* > 0.05) and novel cues (middle panel, Student’s *t*-test, *p = 0.0415) during the generalization test and the generalization indices (right panel, Student’s *t*-test, *p = 0.0146). **e** Freezing levels during extinction training (two-way RM ANOVA followed by Sidak’s post hoc test, *p* > 0.05). Each block represents three CS presentations. **f** Mean freezing levels in response to the CS during the extinction memory test (Mann‒Whitney *U*-test, *p = 0.0271). **g**–**j** Auditory fear conditioning and behavioral data of SST;ErbB4 KO (*n* = 20) and littermate control (*n* = 16) mice. **g** Basal freezing levels during habituation and freezing levels in response to the CS during fear conditioning (two-way RM ANOVA followed by Sidak’s post hoc test, *p* > 0.05). **h** Mean freezing levels in response to the CS (left panel, Mann‒Whitney *U*-test, *p* > 0.05) and novel cues (middle panel, Student’s *t*-test, *p = 0.0415) during the generalization test and the generalization indices (right panel, Student’s *t*-test, *p = 0.0146). **i** Freezing levels during extinction training (two-way RM ANOVA followed by Sidak’s post hoc test, *p* > 0.05). Each block represents three CS presentations. **j** Mean freezing levels in response to the CS during the extinction memory test (Student’s *t*-test, *p = 0.0205). **k**–**n** Auditory fear conditioning and behavioral data for VIP;ErbB4 KO (*n* = 14) and littermate control (*n* = 12) mice. **k** Basal freezing levels during habituation and freezing levels to the CS during fear conditioning (two-way RM ANOVA followed by Sidak’s post hoc test, *p* > 0.05). **l** Mean freezing levels in response to the CS (left panel, Mann‒Whitney *U*-test, *p* > 0.05) and novel cues (middle panel, Mann‒Whitney *U*-test, *p* > 0.05) during the generalization test, and the generalization indices are shown (right panel, Student’s *t*-test, *p* > 0.05). **m** Freezing levels during extinction training (two-way RM ANOVA followed by Sidak’s post hoc test, *p* > 0.05). Each block represents three CS presentations. **n** Mean freezing levels in response to the CS during the extinction memory test (Student’s *t*-test, *p* > 0.05). The data were presented as the means ± SEMs. *p < 0.05, **p < 0.01, and ns not significant.

The ablation of ErbB4 can cause synaptic alterations, which subsequently result in behavioral changes^20,36^. However, the functions of ErbB4 in the modulation of learned fear remain unclear. Therefore, we employed the Cre-lox system for cell type-specific ErbB4 knockout (KO). Female mice with floxed ErbB4 (ErbB4^f/f^) alleles were mated with male mice harboring Cre recombinase driven by the gene of interest (GOI; Dlx5/6, SST, VIP, or PV). The resulting ErbB4^f/f^ double transgenic (GOI^ErbB4-/-^) mice were further bred to generate cell type-specific ErbB4 KO mice and their littermate control mice (Fig. 1a).

To assess the fear responses of those conditional KO mouse lines, we used an auditory fear conditioning paradigm along with fear generalization and extinction memory tests. The mice underwent training with an auditory cue (conditioned stimulus, CS) with electric shocks (conditioned stimuli, US) following 2 consecutive days of habituation. Behavioral analyses of fear generalization, extinction training, and extinction memory were sequentially conducted after fear conditioning (Fig. 1b).

Initially, we generated Dlx5/6^ErbB4-/-^ mice and examined their behaviors to investigate the effect of ErbB4 deletion in all GABAergic neurons. While ErbB4 loss in GABAergic neurons did not affect fear conditioning or extinction training per se (Fig. 1c, e), Dlx5/6^ErbB4-/-^ mice presented an elevated freezing level to a novel cue (NC) and deficits in retrieving extinction memory compared with their littermate controls, indicating apparent fear generalization, as well as compromised extinction (Fig. 1d, f).

GABAergic neurons can be classified into various types, and previous studies have revealed that each cell type plays dedicated roles in the neural circuitry, leading to differential control of fear behaviors^43,44^. Thus, we sought to determine whether ErbB4 loss contributes to the abnormal expression of fear memory that Dlx5/6^ErbB4-/-^ mice displayed. After generating SST-, VIP-, and PV-specific ErbB4 KO mice (Fig. 1a), we performed fear conditioning and subsequently conducted behavioral tests. Compared with their littermate controls, SST^ErbB4-/-^ mice exhibited a heightened fear response to the NC (Fig. 1h) and a higher freezing level to CS during the extinction memory test (Fig. 1j), recapitulating the behavioral phenotypes of Dlx5/6^ErbB4-/-^ mice. However, VIP^ErbB4-/-^ mice displayed levels of fear conditioning, retrieval, and extinction comparable to those of the control mice (Fig. 1k–n). PV^ErbB4-/-^ mice were also analyzed in a slightly different experimental configuration. Compared with their littermate controls, the mice lacking ErbB4 in PV^+^ neurons exhibited decreased freezing in response to the presentation of three initial CSs during extinction training and decreased freezing responses to the CS during the extinction memory test (Supplementary Fig. 1). These results align with those of a previous report indicating that ErbB4 expression in PV^+^ neurons is necessary for fear expression, i.e., the formation of fear memory is impaired by ErbB4 deletion^36^. Taken together, our behavioral data from cell type-specific ErbB4 KO mice indicate that ErbB4 expression in SST^+^ neurons is required for the optimal regulation of fear generalization and extinction memories.

### Deletion of ErbB4 in CeL^SST^ neurons increases anxiety levels and fear generalization

SST^+^ neurons are distributed throughout the brain^45^ and play specific roles in regulating neural circuits for fear memory depending on the brain region in which they reside, from the prefrontal cortex^46^ to the amygdala complex^13,25^. Thus, we attempted to elucidate the physiological and functional impacts of ErbB4 deletion in a region- and cell type-specific manner. We designed ErbB4-targeting sgRNAs to delete ErbB4 in SST^+^ neurons in the regions of interest through the CRISPR/Cas system. Three candidate sgRNA sequences for ErbB4 and a control sgRNA were synthesized and validated. Vectors encoding Cas9 and one candidate sgRNA were transfected into N2A cells known to express ErbB4^47^, and then, the expression of the ErbB4 mRNA was quantified via qRT‒PCR (Supplementary Fig. 2a). The ErbB4 mRNA was rarely detected when sgErbB4v1 was used (Supplementary Fig. 2b). Furthermore, the in vivo efficacy of AAV encoding sgErbB4v1 was verified in SST-Cas9 double transgenic mice (Supplementary Fig. 2c–e). To explore the effects of ErbB4 deletion on synaptic transmission, we also performed whole-cell patch-clamp recordings from sgErbB4v1 virus-injected or sgCtrl virus-injected mice (Supplementary Fig. 2d). The deletion of ErbB4 in CeL^SST^ neurons increased inhibitory synaptic transmission (Supplementary Fig. 2f–i). Thereafter, AAV-sgErbB4v1 was used to delete ErbB4 in SST^+^ neurons in mice

**Fig. 2:**
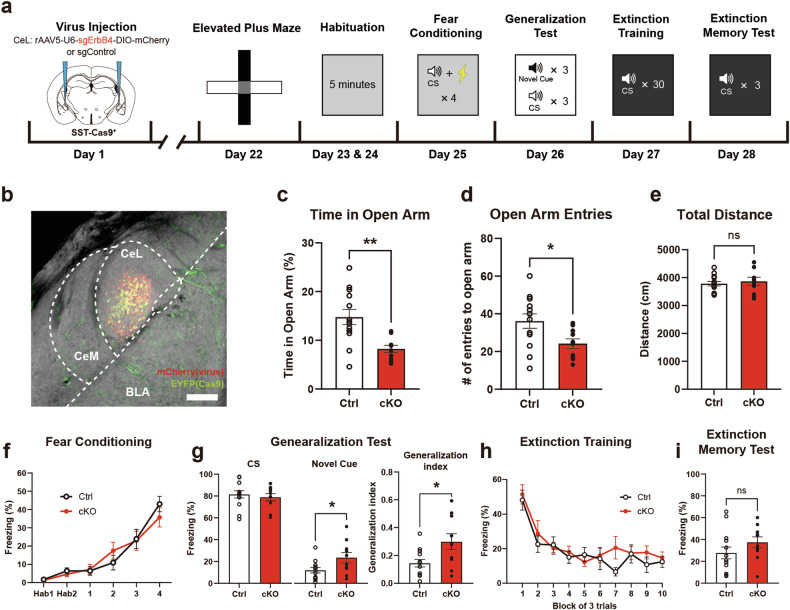
The deletion of ErbB4 in CeL^SST^ neurons increases anxiety and fear generalization. **a** Schematic of the timeline for ErbB4 cKO in CeL^SST^ neurons generated via the CRISPR-Cas9 system and subsequent behavioral tests. **b** A representative merged image indicating a virus-injected site. Scale bar: 200 µm. **c**–**e** Elevated plus maze parameters of the KO virus-injected (cKO; *n* = 10) and control virus-injected (Ctrl; *n* = 13) groups. **c** Mean time spent in the open arms (Student’s *t*-test, **p = 0.0023), **d** mean number of entries into the open arms (Student’s *t-*test, *p = 0.0232), and **e** mean total distance traveled (Student’s *t*-test, *p* > 0.05) in the elevated plus maze. **f**–**i** Auditory fear conditioning and behavioral data for the cKO and Ctrl groups. **f** Basal freezing levels during habituation and freezing levels to the CS during fear conditioning (two-way RM ANOVA followed by Sidak’s post hoc test, *p* > 0.05). **g** Mean freezing levels in response to the CS (left panel, Student’s *t*-test, *p* > 0.05) and novel cues (middle panel, Student’s *t*-test, *p = 0.0303) during the generalization test and the generalization indices (right panel, Student’s *t*-test, *p = 0.0159). **h** Freezing levels during extinction training (two-way RM ANOVA followed by Sidak’s post hoc test, *p* > 0.05). Each block represents three CS presentations. **i** Mean freezing levels in response to the CS during the extinction memory test (Student’s *t*-test, *p* > 0.05). The data were presented as the means ± SEMs. *p < 0.05, **p < 0.01, and ns not significant.

It was previously shown that ErbB4 deletion in CeL^SST^ neurons is sufficient to elevate anxiety levels^20^. Given the substantial overlap between the neural circuits for regulating anxiety states and fear memory^48^, as well as the high comorbidity of anxiety and PTSD^49^, a reasonable hypothesis is that ErbB4 deletion in CeL^SST^ neurons could induce heightened and generalized fear responses, which are endophenotypes of PTSD. We examined this possibility by bilaterally infusing AAV encoding either sgErbB4v1 or the control sgRNA into the CeL of SST-Cas9 mice. The AAV was administered 3 weeks before beginning the behavioral tests, and the elevated plus maze test was subsequently used to assess anxiety levels before fear conditioning (Fig. 2a). Consistent with a previous study^20^, the conditional KO (cKO) mice that received AAV-sgErbB4v1 spent less time in the open arms and had fewer entries into the open arms than the control virus-injected mice did, whereas both groups exhibited comparable locomotion (Fig. 2c–e), indicating increased anxiety when ErbB4 was deleted in CeL^SST^ neurons. However, those cKO mice exhibited freezing levels during both fear conditioning and extinction training that were comparable to those of the control mice (Fig. 2f, h). Importantly, cKO mice showed a significantly higher level of fear generalization (Fig. 2g) and tended to freeze more in the extinction memory test, although freezing levels during the extinction memory test did not differ significantly (Fig. 2i). Overall, ErbB4 expression in SST neurons, specifically in the CeL, is most likely necessary for the proper regulation of anxiety levels and learned fear.

### PTSD-like behavioral traits are correlated with ErbB4 expression in CeL^SST^ neurons

**Fig. 3:**
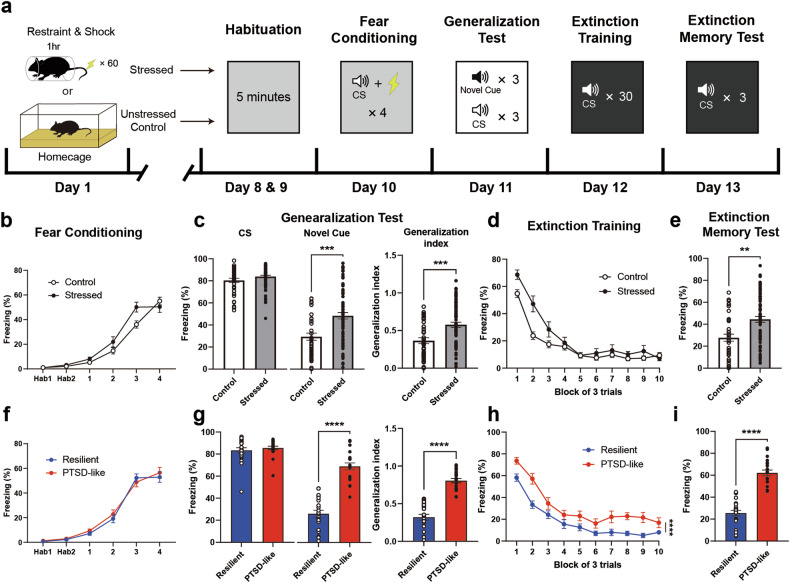
Only a subset of mice exhibit PTSD-like fear responses after stress exposure. **a** Schematic of the timeline depicting stress exposure and subsequent behavioral tests. **b**–**e** Auditory fear conditioning and behavioral data for stressed (*n* = 77) and unstressed control (*n* = 38) mice. **b** Basal freezing levels during habituation and freezing levels to the CS during fear conditioning (two-way RM ANOVA followed by Sidak’s post hoc test, *p* > 0.05). **c** Mean freezing levels in response to the CS (left panel, Mann‒Whitney *U*-test, *p* > 0.05) and novel cues (middle panel, Mann‒Whitney *U*-test, ***p = 0.0003) during the generalization test and the generalization indices (right panel, Student’s *t*-test, ***p = 0.0002). **d** Freezing levels during extinction training (two-way RM ANOVA followed by Sidak’s post hoc test, *p* > 0.05). Each block represents three CS presentations. **e** Mean freezing levels in response to the CS during the extinction memory test (Student’s *t*-test, **p = 0.0025). **f**–**i** Auditory fear conditioning and behavioral data for PTSD-like (*n* = 21) and resilient (*n* = 19) mice after being subjected to stress. **f** Basal freezing levels during habituation and freezing levels to the CS during fear conditioning (two-way RM ANOVA followed by Sidak’s post hoc test, *p* > 0.05). **g** Mean freezing levels in response to the CS (left panel, Mann‒Whitney *U*-test, *p* > 0.05) and novel cues (middle panel, Student’s *t*-test, ****p < 0.0001) during the generalization test and the generalization indices (right panel, Student’s *t*-test, ****p < 0.0001). **h** Freezing levels during extinction training (two-way RM ANOVA followed by Sidak’s post hoc test, ****p < 0.0001). Each block represents three CS presentations. **i** Mean freezing levels in response to the CS during the extinction memory test (Student’s *t*-test, ****p < 0.0001). The data were presented as the means ± SEMs. *p < 0.05, **p < 0.01, ***p < 0.001, ****p < 0.0001, and ns not significant.

Given our observation that appropriate expression of fear memory was sensitive to ErbB4 deletion in CeL^SST^ neurons, we investigated whether ErbB4 expression in CeL^SST^ neurons would be accordingly altered in animals exhibiting abnormal fear responses. We have previously shown that after exposure to traumatic events, mice exhibit elevated fear generalization and deficits in extinction memory^6^, similar to what we detected in SST^ErbB4-/-^ mice. When subjected to excessive stress, consisting of 1 h of restraint stress coupled with 60 electric tail shocks 1 week before the behavioral tests (Fig. 3a), the freezing levels of the mice that were subjected to fear conditioning and extinction training were not different from those of the naïve mice (Fig. 3b, d). Importantly, stressed animals displayed higher freezing to the NC and deficits in extinction memory compared with those of unstressed naïve mice (Fig. 3c, e).

Although all the mice underwent the same stress paradigm, only a subset of these mice displayed abnormal fear responses, whereas the remainder presented fear responses comparable to those of unstressed naïve mice. Therefore, we classified those subject mice that failed to discriminate the NC from the CS and exhibited impaired fear extinction memory as “PTSD-like” animals since PTSD models exhibit such behavioral traits^8,10,11^. Conversely, mice that managed to regulate fear responses despite exposure to excessive stresses comparable to those of unstressed mice were classified as “resilient” animals. For the systematic classification of groups, we used the k-means clustering method with behavioral data from fear generalization and extinction memory tests (Supplementary Fig. 3)^6^. As expected, the PTSD-like group displayed greater fear generalization (Fig. 3g) and higher freezing levels during extinction training (Fig. 3h) and even after extinction training (Fig. 3i) than a resilient group. However, freezing levels did not differ between groups during fear conditioning (Fig. 3f).

**Fig. 4:**
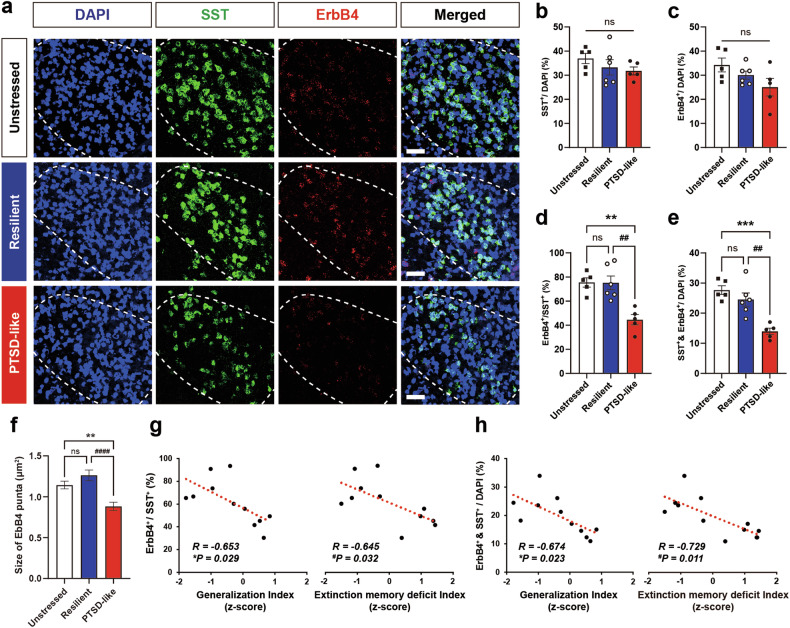
PTSD-like fear responses are inversely correlated with ErbB4 expression in CeL^SST^ neurons. **a** Representative images of SST and ErbB4 expression in the CeL of unstressed control (*n* = 5), resilient (*n* = 6), and PTSD-like (*n* = 5) mice. CeL areas are outlined with white dotted lines. Blue, DAPI; green, SST; red, ErbB4. Scale bars: 50 µm. **b**–**e** Cell counts for DAPI-stained, SST^+^, and ErbB4^+^ cells in the CeL. **b** Percentages of SST^+^ cells among DAPI-stained cells (one-way ANOVA with Tukey’s post hoc test, *p* > 0.05). **c** Percentages of ErbB4^+^ cells among DAPI-stained cells (one-way ANOVA with Tukey’s post hoc test, *p* > 0.05). **d** Percentages of ErbB4^+^ cells among SST^+^ cells (one-way ANOVA with Tukey’s post hoc test, ***p = 0.0003 and ^##^*p* = 0.0020). **e** Percentages of ErbB4- and SST-coexpressing cells among DAPI-stained cells (one-way ANOVA with Tukey’s post hoc test, **p = 0.0021 and ^##^*p* = 0.0016). **f** Quantification of the average size of ErbB4 puncta in SST+ cells (one-way ANOVA with Tukey’s post hoc test, **p = 0.0034 and ^####^*p* < 0.0001). **g** Scatter plots displaying correlations between the ratios of ErbB4^+^/SST^+^ cells and the generalization indices (left panel) and the ratios of ErbB4^+^/SST^+^ cells and the extinction memory deficit indices (right panel) in PTSD-like and resilient mice (Pearson’s correlation coefficients, *R* = −0.653, *p = 0.029, and *R* = −0.629, ^#^*p* = 0.038). **h** Scatter plots displaying correlations between the ratios of ErbB4^+^ and SST^+^/DAPI cells and the generalization indices (left panel) and between the ratios of ErbB4^+^ and SST^+^/DAPI cells and the extinction memory deficit indices (right panel) in PTSD-like and resilient mice (Pearson’s correlation coefficients, *R* = −0.674, *p = 0.023, and *R* = −0.710, ^#^*p* = 0.014). The data were presented as the means ± SEMs. *p < 0.05, **p < 0.01, ***p < 0.001, and ns not significant.

To explore any possible correlation between PTSD-like fear responses and ErbB4 expression in CeL^SST^ neurons, we used FISH to quantify the abundance of SST- and ErbB4-expressing neurons within the CeL area in PTSD-like, resilient, and unstressed naïve mice. The ratios of SST-expressing neurons and ErbB4-expressing neurons to total DAPI-labeled cells did not differ across the groups (Fig. 4a–c). Importantly, the proportions of ErbB4^+^ neurons among SST^+^ neurons and CeL neurons coexpressing SST and ErbB4 were lower in the PTSD-like group than in the unstressed or resilient groups. The average size of ErbB4 puncta in SST^+^ neurons was also smaller in the PTSD-like group. Conversely, these proportions and ErbB4 puncta sizes did not differ between the unstressed and resilient groups (Fig. 4d–f). Furthermore, the severity of the PTSD-like phenotypes, represented by indices of fear generalization and extinction memory deficits, was negatively correlated with the proportion of ErbB4-expressing CeL^SST^ neurons (Fig. 4g, h and Supplementary Table 1). These results support the possibility that ErbB4 could prevent animals from evoking PTSD-like traits after stressful experiences.

### ErbB4 in SST^+^ neurons is required to maintain the activity selectivity of CeL neurons

**Fig. 5:**
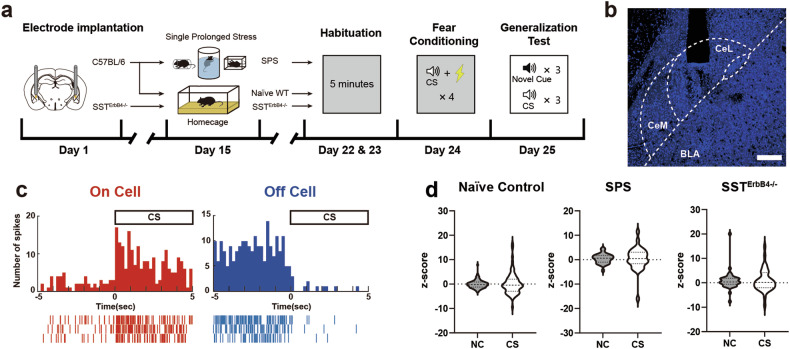
Bimodal activity of CeL neurons in response to the CS and novel cues. **a** Schematic of the timeline for chronic electrode implantation, stress exposure, and subsequent behavioral tests. **b** A chronic electrode implantation site in the CeL. Scale bar: 200 µm. **c** Example peristimulus time histograms (top panels) and raster plots (bottom panels) of an On cell (red, left panels) and an Off cell (blue, right panels) in response to the CS during the generalization test. **d** Violin plots of the distribution of neuronal responses to the CS and NC. Units from all groups exhibited stronger bimodal responses to the CS than to the NC. The standard deviations of unit activity are as follows: naïve control (NC: 1.716, CS: 4.377), SPS (NC: 1.951, CS: 4.676), and SST^ErbB4-/-^ (NC: 3.863, CS: 4.472). Dashed lines: medians; dotted lines: quartiles.

Given that SST^ErbB4-/-^ mice exhibited PTSD-like fear responses, the neuronal activity of the CeL could mediate the operational actions of ErbB4 and thereby underlie the behavioral changes that SST^ErbB4-/-^ mice exhibited. We performed in vivo single-unit recordings from CeL neurons during the generalization sessions from SST^ErbB4-/-^ and littermate control mice to address this notion. We implanted custom-made electrodes into the CeL region 3 weeks before the commencement of the behavioral experiments (Fig. 5a, b). As previously reported^16,22^, a subset of neurons displayed excitatory responses to the CS, whereas another subset of neurons showed inhibitory responses (Fig. 5c). The units recorded from CeL neurons displayed stronger bimodal responses to the CS than the NC across all groups (Fig. 5d). We further classified the units into three types: “On cells”, “Off cells”, and nonresponsive (NR) cells. When the unit activity to the CS and NC was z score normalized relative to their own baseline activity obtained during the initial 3 min of acclimation, the units that showed mean activity to stimuli with z scores of greater than 1 or less than −1 were designated On and Off cells, respectively. The remaining units that did not meet these criteria were categorized as NR cells.

**Fig. 6:**
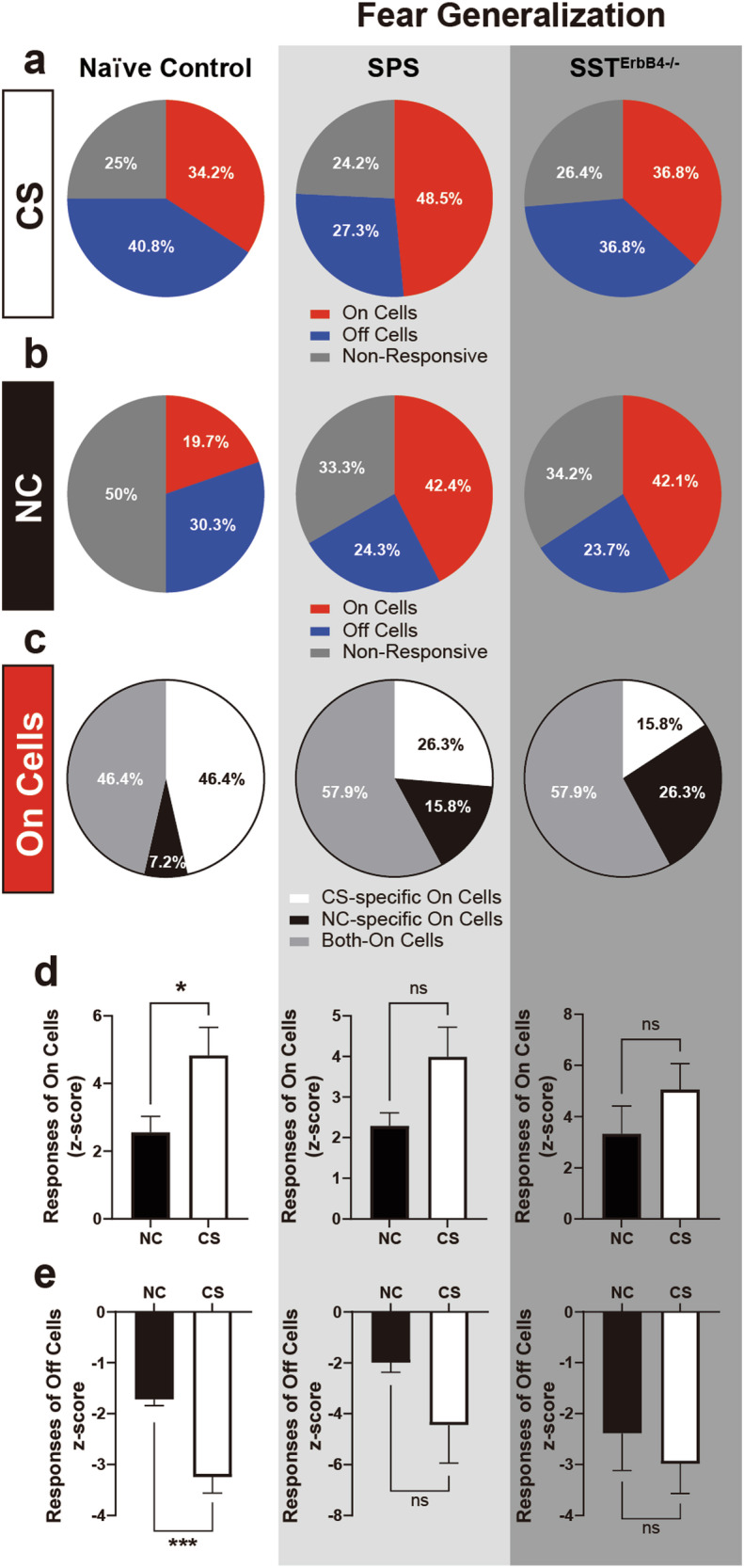
ErbB4 in SST^+^ neurons is required for the bimodal activity of CeL On/Off cells. **a**, **b** Proportions of On, Off, and nonresponsive cells in naïve control (*N* = 14 mice, *n* = 76 units, white background), SPS (*N* = 8 mice, *n* = 32 units, gray background), and SST^ErbB4-/-^ (*N* = 10 mice, *n* = 38 units, dark gray background) mice in response to the CS (**a**) and NC (**b**). The proportions of On, Off, and nonresponsive cells in response to the CS were comparable across all groups (chi-square test, *p* > 0.05). However, in response to the NC, the proportions were significantly different between the naïve control and SPS groups (chi-square test, *p = 0.0458) and between the naïve control and SST^ErbB4-/-^ groups (chi-square test, *p = 0.0395). **c** Proportion of On cells responding to the CS, NC, or both in naïve control (*n* = 28 units), SPS (*n* = 19 units), and SST^ErbB4-/-^ (*n* = 19 units) mice. The proportion of On cells was significantly different only between the naïve control and SST^ErbB4-/-^ groups (chi-square test, *p = 0.0449). **d** Mean z scores of the responsive activity of On cells to the NC and CS in the naïve control (left panel, Mann‒Whitney *U*-test, *p = 0.0439), SPS (middle panel; Mann‒Whitney *U*-test, *p* > 0.05), and SST^ErbB4-/-^ (right panel; Mann‒Whitney *U*-test, *p* > 0.05) groups. **e** Mean z scores of the responsive activity of Off cells to the NC and CS in the naïve control (left panel, Mann‒Whitney *U*-test, ***p = 0.0003), SPS (middle panel; Mann‒Whitney *U*-test, *p* > 0.05), and SST^ErbB4-/-^ (right panel; Mann‒Whitney *U*-test, *p* > 0.05) groups. The data were presented as the means ± SEMs. *p < 0.05, **p < 0.01, ***p < 0.001, and ns not significant.

We further adopted the single prolonged stress (SPS) protocol for stress administration^38,50^ to further simulate traumatic events in PTSD patients and experimentally increase the fraction of animals with PTSD phenotypes (SPS group). As expected, the SPS and SST^ErbB4-/-^ groups exhibited potent fear generalization (Supplementary Fig. 4). The proportions of each cell type, On, Off and NR cells, were comparable across the three groups when the CS was presented (Fig. 6a). Interestingly, the proportions of On cells responding to the NC increased in the SPS (42.4%) and SST^ErbB4-/-^ groups (42.1%) compared with those in the naïve control group (19.7%, Fig. 6b). When only On cells were pooled, the proportions of CS-specific On cells were substantially lower in the SPS (26.3%) and SST^ErbB4-/-^ (15.8%) groups than in the naïve control group (46.4%, Fig. 6c). Conversely, the number of Both-On cells, which responded to both the CS and the NC, was slightly greater in the SPS (57.9%) and SST^ErbB4-/-^ (57.9%) groups than in the naïve control group (46.4%, Fig. 6c). We also assessed the intensity amplitudes from On cells to the CS and NC. While the responses of On cells to the CS in the naïve control group were greater than those to the NC, the responses of On cells to the CS did not differ from those to the NC in the SPS and SST^ErbB4-/-^ groups (Fig. 6d). Additionally, the activity of Off cells in response to the NC was significantly lower than that in response to the CS in the naïve control group but not in the SPS and SST^ErbB4-/-^ groups (Fig. 6e). Overall, the overall trends in the compositional changes in and differences in the activity of On/Off cells between the CS and NC observed in the SPS and SST^ErbB4-/-^ mice paralleled their behavioral characteristics, i.e., fear generalization. Therefore, these behavioral data support the possibility that ErbB4 expressed in SST^+^ neurons is necessary for the maintenance of the CS-specific bimodal activity of CeL neurons, which is likely to foster the optimal discrimination of ambient stimuli.

## Discussion

In the present study, we used Dlx5/6-, SST-, VIP-, and PV-specific ErbB4 KO mice to systematically examine the behavioral and physiological roles of ErbB4 in fear-related behaviors, including fear conditioning, fear generalization, and extinction (Fig. 1). ErbB4 deletion in GABAergic neurons, especially in CeL^SST^ neurons, was sufficient to induce PTSD-like phenotypes (Fig. 2), suggesting preventive roles of ErbB4 in CeL^SST^ neurons against the occurrence of PTSD-like behaviors following exposure to traumatic events.

In this study, we adopted restraint and electric shock as traumatic stresses to evoke PTSD-like behavior. We observed no significant effect of this stress on freezing during fear conditioning (Fig. 3), which contrasts with some reports of a stress-induced enhancement of fear learning^51,52^. However, this discrepancy is not unusual, as several studies have also failed to observe such an enhancement^6,53,54^. The variability in outcomes can be attributed to differences in experimental designs and methods used for analyzing freezing behavior. Despite these variations, the general trend of stress-induced increases in fear expression across studies supports the use of stress paradigms as effective models for inducing PTSD-like phenotypes in mice.

Our FISH experiments for SST and ErbB4 in the CeL revealed a notable decrease in ErbB4 expression in SST neurons in stressed animals displaying PTSD-like phenotypes (Fig. 4d, e). Stress-induced apoptosis in the amygdala is increased in PTSD models^5^, and the volume of the amygdala complex is decreased in PTSD patients^55^. This atrophy might result from increased death of amygdala neurons due to the overexcitation of amygdala neural circuits caused by downregulated ErbB4 activity because ErbB4 is required for the promotion and maintenance of the inhibitory drive^30–32^. We detected that the expression of either ErbB4 or SST remained comparable across groups, but the abundance of ErbB4-expressing SST^+^ neurons decreased only in the PTSD-like group (Fig. 4). In the present study, we were unable to elucidate how the abundance of SST^+^ neurons expressing ErbB4 in the CeL decreased in animals that displayed PTSD-like phenotypes. Thus, the reduction in ErbB4-expressing SST^+^ neurons that we observed in stressed animals reflects the interplay between ErbB4 and SST and the increased sensitivity of CeL neurons to stressors, potentially due to elevated levels of corticosterone.

Consistent with previous studies^16,22^, we validated and identified On/Off cells in the CeL that respond to a CS or NC. Similar to the diminished responsiveness to the CS after fear extinction^22^, our recordings from naïve control mice also revealed lower responses of On/Off cells to the NC than to the CS (Fig. 6d, e). Notably, the On/Off cells of the SPS and SST^ErbB4-/-^ groups displayed magnitudes of activity that were comparable in response to the CS and NC. Fear generalization may result from the generalized activity of CeL On/Off cells when ErbB4 is deleted in CeL^SST^ neurons and when animals are subjected to intense stress. Therefore, the nonselective activity of On/Off cells is likely to underlie the generalized expression of fear memory that we observed in SPS and SST^ErbB4-/-^ mice. In fact, the administration of NRG1 has been shown to promote fear extinction^56^ and alleviate depression-like behaviors caused by chronic defeat stresses^57^. These observations suggest that ErbB4 is a novel therapeutic target for the treatment of stress disorders, including PTSD. Moreover, because SST^ErbB4-/-^ mice exhibit PTSD-like behaviors with the generalized activity of CeL On/Off cells, SST^ErbB4-/-^ mice constitute a mechanism-based PTSD animal model that can recapitulate the neuronal and behavioral features of PTSD.

Using whole-cell patch-clamp recordings, we confirmed that ErbB4 deletion in CeL^SST^ neurons resulted in an increase in inhibitory synaptic transmission (Supplementary Fig. 2f–i). However, ErbB4 deletion in CeL^SST^ neurons was previously shown to increase excitatory inputs^20^. Considering that the SST^ErbB4-/-^ mice exhibited enhanced fear responses (Fig. 1g–j) and that On cells in these mice showed stronger responses to novel cues during the generalization test (Figs. 5d, 6d), ErbB4 deletion in CeL^SST^ neurons might lead to an increase in neuronal activity. This scenario is consistent with the established role of CeL^SST^ neurons and On cells that mediate fear responses^16,22,23,25^. Further studies with in vivo activity measurements are needed to answer this question.

This study revealed that ErbB4 expression in the CeL can control behavioral and neuronal fear responses after exposure to stress, leading to maladaptive PTSD-like responses. Our observation of PTSD-like phenotypes in ErbB4 KO mice also underscores the significant role of ErbB4-NRG1 through pivotal mechanisms that modulate emotional responses, particularly after a traumatic experience. Importantly, this study provides experimental evidence clarifying the previously unknown physiological roles of ErbB4 in CeL On/Off cells and suggesting a potential mechanism for PTSD. The observed function of ErbB4 expressed in CeL^SST^ neurons in PTSD-like behaviors not only sheds light on the cellular and circuit mechanisms of PTSD-like behaviors but also indicates that ErbB4 is a promising therapeutic and diagnostic target for PTSD.

## Supplementary information

SUPPLEMENTAL MATERIAL
