# Supplementary material for: ErbB4 precludes the occurrence of PTSD-like fear responses by supporting the bimodal activity of the central amygdala
Source: Exp Mol Med. 2024 Dec 2;56(12):2703–13. doi: 10.1038/s12276-024-01365-1 (PMC11671592; doi:10.1038/s12276-024-01365-1)
Supplement: Supplementary file 1 — SUPPLEMENTAL MATERIAL [file 12276_2024_1365_MOESM1_ESM.pdf]

## Supplementary Information

# ErbB4 precludes the occurrence of PTSD-like fear responses by supporting the bimodal activity of the central amygdala

### Supplementary Figures and Table

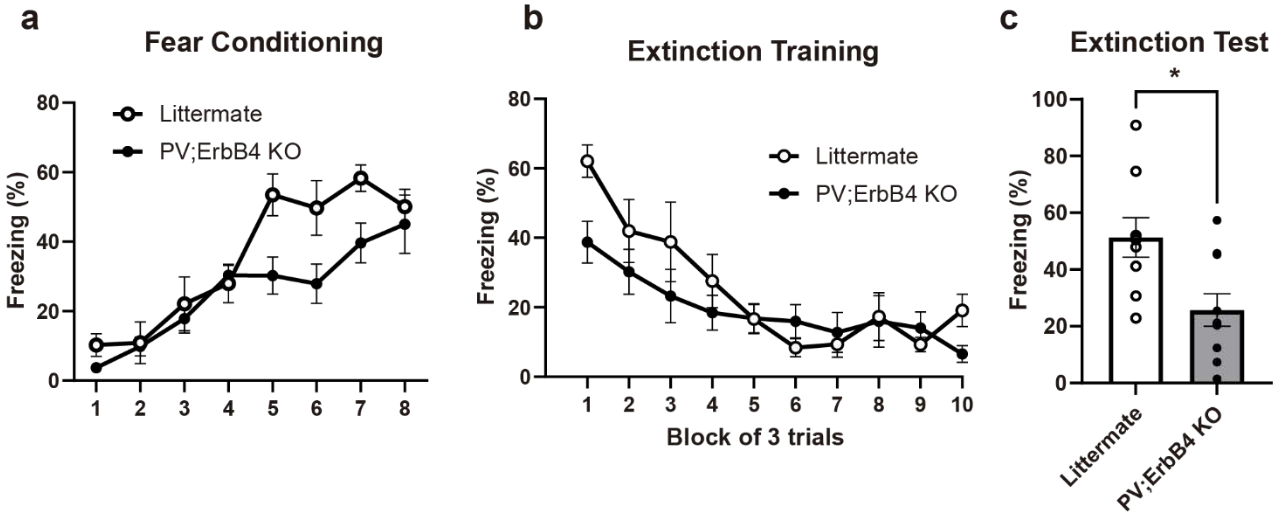


**Supplementary Fig. 1: Deletion of ErbB4 in the parvalbumin (PV)-positive neurons disrupts learned fear responses.**

**a–c** Auditory fear conditioning and behavioral data of PV;ErbB4 KO (n = 10) and their littermate control (n = 9) mice with eight CS-US paring paradigms. (**a**) Freezing levels to CS during fear conditioning (two-way RM ANOVA followed by Sidak's post hoc test, $p > 0.05$). (**b**) Freezing levels during extinction training (two-way RM ANOVA, $p > 0.05$). Each block represents three CS presentations. (**c**) Mean freezing levels to CS during the extinction memory test (Student's t-test, *$p = 0.0107$). The data are expressed as the mean ± SEM. *$p < 0.05$.

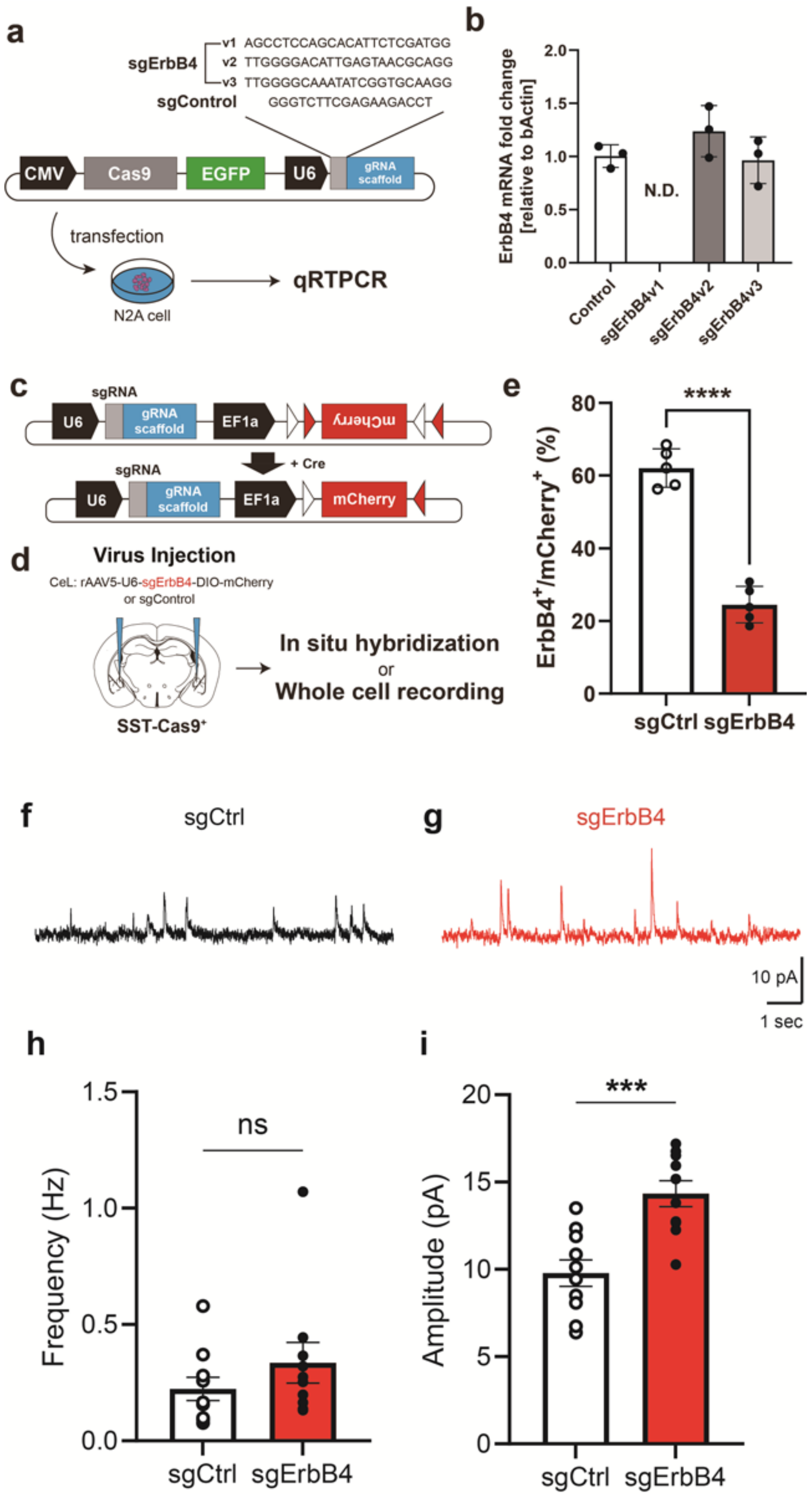


**Supplementary Fig. 2: Screening and validation of ErbB4-targeting sgRNA.**

**(a)** Illustration for ErbB4-targeting sgRNA screening using Neuro-2a cells. **(b)** The effectiveness of ErbB4-targeting sgRNA candidates. ErbB4 mRNA is not detected in sgErbB4v1-transfected cells (one-way ANOVA with Tukey's post hoc test) **(c)** A diagram of sgErbB4v1-encoding vector used for ErbB4 deletion. **(d)** A schematic for *in vivo* validation of ErbB4-targeting sgRNA (sgErbB4). The virus is injected 3 weeks before FISH. **(e)** The ratios of FISH-stained ErbB4-postive neurons among all virally-infected (mCherry$^+$) CeL cells (Student's t-test,

****$p < 0.0001$). **f-i** Miniature IPSCs (mIPSCs) from virally-infected CeL cells of the control virus-injected (N=2, n=10) and the KO virus-injected mice (N=2, n=10). The representative traces of mIPSCs from sgCtrl group **(f)** and sgErbB4 group **(g)** are presented. **(h)** Frequency of mIPSCs from sgCtrl and sgErbB4 groups (Mann-Whitney U-test, $p>0.05$) and **(i)** amplitude of mIPSCs from sgCtrl and sgErbB4 groups are presented (Student's t-test, ***$p = 0.0004$). The data are expressed as the mean ± SEM. *$p < 0.05$, **$p < 0.01$, ***$p < 0.001$, ****$p < 0.0001$, ns, not significant, N.D., not detected.

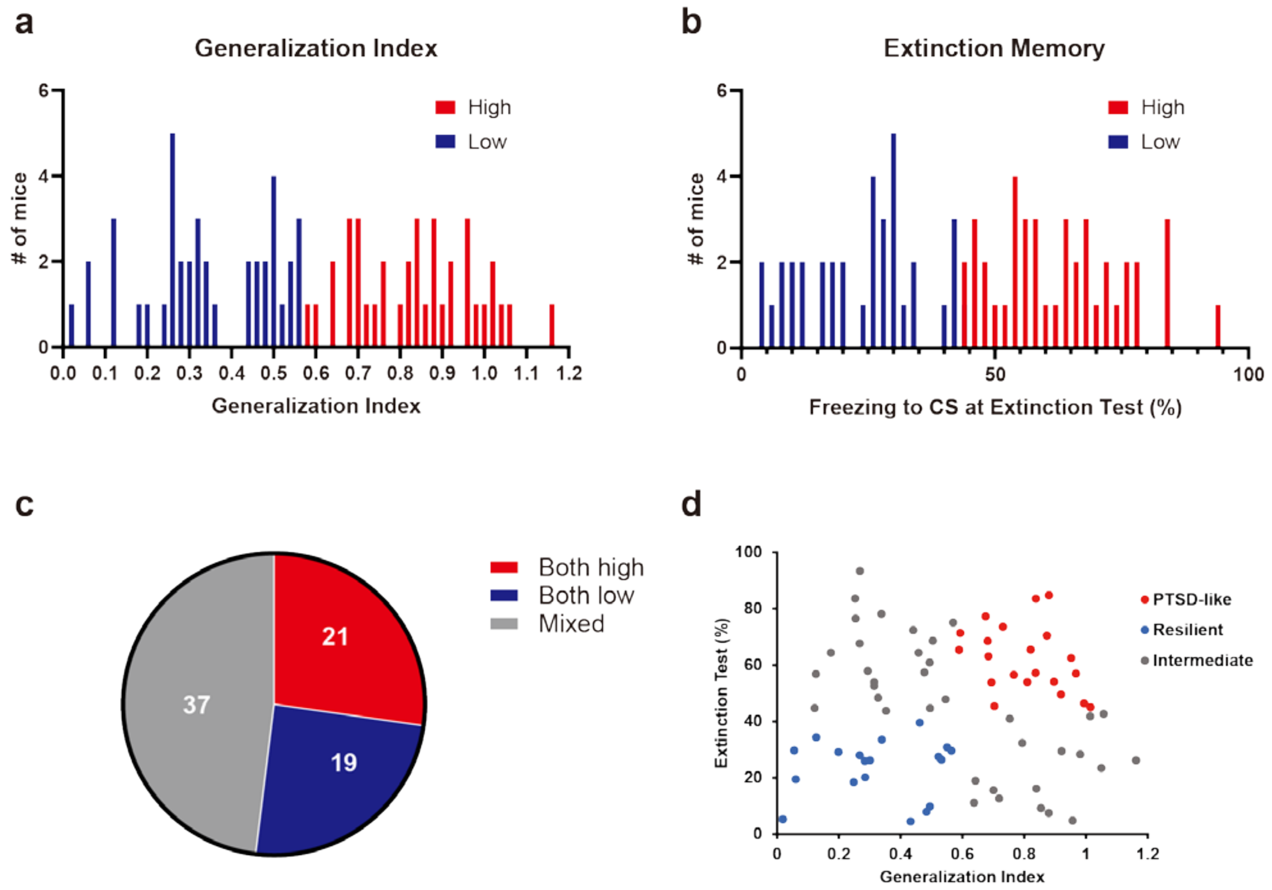


**Supplementary Fig. 3: Classification of stressed mice into PTSD-like and resilient groups via k-means clustering.**

**(a)** K-means clustering analysis of the generalization indices of stressed mice (n = 77). Red, high (n = 37 mice); blue, low (n = 40 mice). **(b)** K-means clustering analysis of freezing levels during extinction tests of stressed mice (n = 77). Red, high (n = 41 mice); blue, low (n = 36 mice). **(c)** Using k-means clustering analysis of results from generalization and extinction tests, individual mice are categorized as follows: “Both high” (high in both; n = 21, 27.27%), “Both low” (low in both; n = 19, 24.68%), and “Mixed” (not belonging to either category; n = 37, 48.05%). Those mice classified as “Both high” are referred to as “PTSD-like” animals, whereas those categorized as “Both low” are referred to as “Resilient”. **(d)** A scatter plot displaying stressed mice using their generalization and extinction memory deficit indices. Red, PTSD-like (n = 21 mice); blue, resilient (n = 19 mice); gray, intermediate (n = 37 mice).

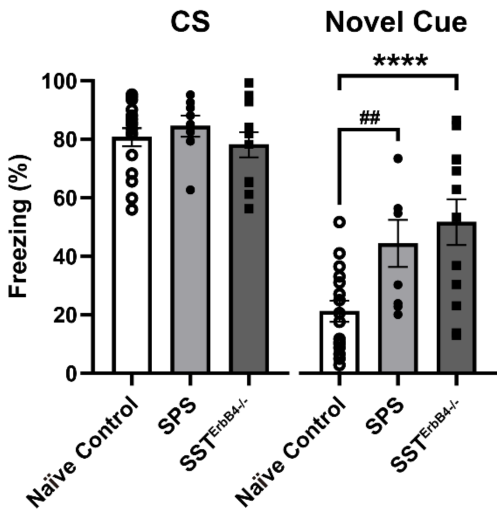


**Supplementary Fig. 4: Fear generalization of recorded mice.**

Fear generalization results of naïve control, SPS and $SST^{ErbB4-/-}$ mice in which *in vivo* activity is monitored. Mean freezing levels to CS (left) and to novel cues (right) are summarized (two-way ANOVA with Sidak's post hoc test, $^{##}p = 0.0097$, $^{****}p < 0.0001$). The data are expressed as the mean ± SEM. $^{*}p < 0.05$, $^{**}p < 0.01$, $^{***}p < 0.001$, $^{****}p < 0.0001$, ns, not significant.

| **Pearson Correlation** | Generalization index (z-score) | | Extinction memory deficit index (z-score) | |
|---|---|---|---|---|
| | Correlation coefficient | P-value | Correlation coefficient | P-value |
| SST/DAPI (%) | −0.12608 | 0.7118 | −0.27457436 | 0.4139 |
| ErbB4/DAPI (%) | −0.33734 | 0.3103 | −0.21636081 | 0.5228 |
| Sst&ErbB4/DAPI (%) | −0.67473 | *0.0227 | −0.72889478 | *0.0109 |
| ErbB4/SST (%) | −0.65342 | *0.0292 | −0.6447944 | *0.0322 |

**Supplementary Table 1. Correlation coefficients and p-values between FISH and fear behavior results.**
